# The Management of Heavy Menstrual Bleeding After Percutaneous Coronary Intervention in a Woman of Reproductive Age

**DOI:** 10.3389/fphar.2018.01573

**Published:** 2019-01-15

**Authors:** Zhi-Chun Gu, Fang-Hong Shi, Jie Zhu, Fang Wan, Long Shen, Hao Li

**Affiliations:** ^1^Department of Pharmacy, Renji Hospital, School of Medicine, Shanghai Jiao Tong University, Shanghai, China; ^2^Department of Obstetrics and Gynecology, Renji Hospital, School of Medicine, Shanghai Jiao Tong University, Shanghai, China; ^3^Department of Cardiology, Renji Hospital, School of Medicine, Shanghai Jiao Tong University, Shanghai, China; ^4^Department of Pharmacy, Shanghai Children’s Medical Center, School of Medicine, Shanghai Jiao Tong University, Shanghai, China

**Keywords:** heavy menstrual bleeding, menorrhagia, reproductive age, acute coronary syndromes, uremia, systemic lupus erythematosus, antiplatelet therapy

## Abstract

Heavy menstrual bleeding (HMB), previously known as menorrhagia, is in place with heavy flow and longer lasting days of bleeding during menstrual period, sequentially leading to anemia. We reported a rare case of HMB in a 33-year-old patient after percutaneous coronary intervention (PCI), who presented with acute coronary syndromes (ACS), uremia and systemic lupus erythematosus before PCI. This patient received three times of hemodialysis weekly (Monday, Wednesday, and Friday). On the next day after PCI, this patient began to have menstruation. On the fifth day of menstruation, the patient complained of HMB and physical discomfort, with an urgent need for consultation of gynecologist. After gynecologist consultation, this patient was under oxytocin treatment. However, 2 days of oxytocin treatment did not significantly improve HMB. Afterward, the menstrual volume of patients was significantly reduced on eighth day of menstruation after once therapy of testosterone propionate and norethindrone. Regarding the reasons of HMB, heparin in hemodialysis and antiplatelet drugs utilized (aspirin and clopidogrel) after PCI may be contributors to the HMB. In addition, uterine myoma, cervical canal cyst, renal insufficiency and *CYP2C19^∗^2* heterozygous are also possible contributors to HMB. There is no such case of whom had HMB in reproductive age with ACS, uremia and systemic lupus erythematosus under hemodialysis and antiplatelet therapy. More clinical safety data on HMB of reproductive age women who are under antithrombotic therapy are required.

## Background

From 1995 to 2010, the prevalence of ST-elevation myocardial infarction increased from 11.8 to 25.5% particularly in women younger than 60 years (Puymirat et al., 2012). As a growing number of premenopausal female patients with various cardiovascular indications, a need of anticoagulants and/or antiplatelet agents has gradually increased (Maas et al., 2015). Women taking antithrombotic therapy may experience both increased volume and duration of menstrual bleeding (Munro et al., 2011). Even though uterine structural or endocrine abnormalities are also related to AUB, the prevalence of HMB in premenopausal women under age 50 years increased from 17.8% before antithrombotic treatment to 29.5% thereafter (Sjalander et al., 2007). Antiplatelet therapy of aspirin and clopidogrel could also lead to AUB and HMB (Maas et al., 2015). The lacks of asking menstrual problems by the majority of cardiologist or vascular specialists in premenopausal female patients may exacerbate the risk of AUB and HMB. We report one such rare case of HMB in a 33-year-old patient after hemodialysis and antiplatelet therapy, who suffers from ACS, uremia and systemic lupus erythematosus.

## Case Presentation

**FIGURE 1:**
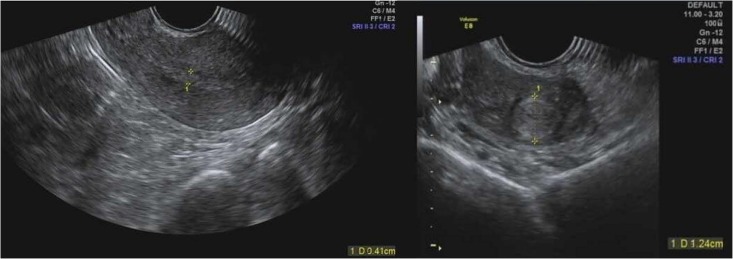
Uterus ultrasound pictures.

Our patient is a 33-year-old women suffering from ACS, uremia and systemic lupus erythematosus. The patient was hospitalized on February 26th 2018 for ACS. Before the introduction of PCI, she had been treated with hemodialysis weekly at Monday, Wednesday, and Friday for years (Table 1). At hospital admission, laboratory testing showed normal hemoglobin level of 116 g/L (Table 1). After PCI on February 26th, 2018, the patient had received aspirin 100 mg q.d. and clopidogrel 75 mg q.d. since February 26th, 2018. On February 27th, 2018, the patient had menstrual bleeding. Due to the presence of increased volume of menstrual bleeding on the fifth day, the patient had extreme discomfort and anxiety, requiring a gynecologic consultation. The reduction of RBC count, hemoglobin and hematocrit leaded to a doubt of normocytic hypochromic anemia (Table 1). Since March 3rd, 2018, the patient received oxytocin 20 IU q.6.h., which could reduce uterine bleeding. However, the HMB had not improved until March 5th 2018. Under the consultation of gynecologists and clinical pharmacists, we investigated whether the uterus was abnormal by using the gynecologic ultrasound, and the results suggested the presence of uterine myoma and cervical canal cyst (Figure 1). Testosterone propionate 25 mg q.d. and norethindrone 5 mg q.8.h. were suggested. After the administration of testosterone propionate and norethindrone, the menstrual volume of the patient was significantly reduced. This patient was discharged from hospital in March 7th, 2018. The patient medications during hospitalization were: rosuvastatin calcium tablets, mosapride citrate dispersible tablets, pantoprazole sodium enteric-coated capsules, compound glycyrrhiza oral solution, and cefdinir dispersible tablets.

**Table 1:** Clinical information and history.

(Normal range)	February 26th 2018	February 28th 2018	February 28th 2018	March 2nd 2018	March 3rd 2018	March 5th 2018	March 7th 2018

Events	PCI First day of menstrual bleeding			Complained of HMB		Start hormone therapy	HMB significantly reduced
**Hematology testing**							
RBC (3.68–5.12 × 10^12^/L)	3.93	-	-	-	3.6 ↓	3.2 ↓	-
WBC (3.69–9.16 × 10^9^/L)	6.50	-	-	-	5.72	6.57	-
N % (50–70 %)	90.9 ↑	-	-	-	72.3 ↑	88.8↑	-
Lymphocyte % (20–40%)	7.4 ↓	-	-	-	19.2 ↓	7.8↓	-
Monocyte % (3–10%)	1.2 ↓	-	-	-	7.0	2.6↓	-
Eosinophils % (0.5–5.0%)	0.2 ↓	-	-	-	1.0	0.5	-
Basophil % (0.0–1.0%)	0.3	-	-	-	0.5	0.3	-
Hemoglobin (113–151 g/L)	116	-	-	-	111 ↓	94 ↓	-
Hematocrit (0.37–0.480 L/L)	0.365 ↓	-	-	-	0.332 ↓	0.295 ↓	-
MCV (82.6–99.1 fl)	92.9	-	-	-	92.2	92.2	-
MCH (26.9–33.3 pg)	29.5	-	-	-	30.8	29.4	-
MCHC (322–362 g/L)	318 ↓	-	-	-	334	319 ↓	-
Glucose (3.9–6.1 mmol/L)	3.34 ↓	-	-	-	-	-	-
HbA1c (4–6%)	4.7	-	-	-	-	-	-
Triglycerides (<1.7 mmol/L)	1.83 ↑	-	-	-	-	-	-
Total cholesterol (<5.72 mmol/L)	5.43	-	-	-	-	-	-
LDL	2.77	-	-	-	-	-	-
HDL	1.59	-	-	-	-	-	-
**Markers of kidney function**							
Creatinine (45–104 μmol/L)	1095.0 ↑	-	-	-	-	1081.0 ↑	-
Urea nitrogen (2.9–8.2 mmol/L)	28.90↑	-	-	-	-	25.50 ↑	-
**Hemodialysis**							
Duration of hemodialysis (hours)	3	4	3	4	-	-	4
Ultrafiltration (mL)	1030	2400	2900	4000	-	-	2700
**Markers of myocardial infarction**							
CK (30–170 U/L)	77	-	-	-	-	-	-
TNI-A2 (<0.04 ng/mL)	6.59 ↑	-	-	-	-	-	-
BNP (0.0–100 pg/mL)	321.0 ↑	-	-	-	-	-	-
PTH (12–88 pg/mL)	1004.5 ↑	-	-	-	-	-	-
CRP (0–3 mg/L)	1.90	-	-	-	-	-	-
**Blood coagulation series detection**							
Fibrin degradation products (0–5 μg/mL)	1.2	-	-	-	-	-	-
Thrombin time (14–21 s)	18.1	-	-	-	16.20	-	-
Prothrombin time (9.4–12.5 s)	9.90	-	-	-	10.40	-	-
Fibrinogen (2.00–4.00 g/L)	2.96	-	-	-	3.53	-	-
Partial thromboplastin (20–40 s)	29.1	-	-	-	28.20	-	-
Prothrombin INR (0.8–1.15)	0.85	-	-	-	0.91	-	-
D-dimer (0–0.5 DDU μg/mL)	0.13	-	-	-	-	-	-


*RBC, red blood cell count; WBC, white blood cell; N %, neutrophil %; MCV, mean corpuscular volume; MCH, mean corpuscular hemoglobin mch; MCHC, mean corpuscular hemoglobin concentration; HbA1c, hemoglobin A1c; LDL, low-density lipoprotein; HDL, high-density lipoproteins; CK, creatine kinase; TNI, troponin I; BNP, brain natriuretic peptide; PTH, parathyroid hormone; CRP, C-reactive protein. ↓, lower than normal value; ↑, higher than normal value.*

## Investigations

Clinical investigations were performed to assess for the causes of HMB at aspirin and clopidogrel therapeutic doses. They included *CYP2C19*, *ABCB1*, *PEAR1*, and *PON1* genotyping, and thromboelastogram.

### *CYP2C19*, *ABCB1*, *PEAR1*, and *PON1* Genotyping

Blood samples were collected before the use of clopidogrel, which were drawn into a vacutainer tube containing 3.8% trisodium citrate. The presence of *CYP2C19^∗^2* (rs4244285), *CYP2C19^∗^3* (rs4986893) was detected via DFMH method using a commercial kit from Sino Era Genotech^1^ (Beijing, China). 200 μL of whole blood was added to 1 mL of RBC lysis buffer and incubated for 5 min at indoor temperature. Centrifuged (3,000 rpm) the mixtures for 5 min then removed the supernatant. Afterward, 50 μL of preservation solution was added to the precipitate. 1.5 μL of sample was combined with detecting reagent that includes hybridization buffer and two molecular beacon probes labeled with FAM and HEX for wild-type or mutation. TL998A real-time PCR system (Tianlong, Xi’an, China) was used to perform the analysis. The detected process began with denaturation for 5 min at 95°C followed by 55 cycles of denaturation for 30 s at 95°C and hybridization for 75 s at 62°C. Based on *CYP2C19* genotype, the patients were classified into three metabolizers, which were extensive metabolizer (EM; ^∗^1/^∗^1), intermediate metabolizer (IM; ^∗^1/^∗^2 or ^∗^1/^∗^3), or poor metabolizer (PM; ^∗^2/^∗^2, ^∗^2/^∗^3, or ^∗^3/^∗^3). *ABCB1* (rs1045642), *PEAR1* (rs12041331), and *PON1* (rs662) genotyping were also detected via DFMH method. Results indicated only a *CYP2C19^∗^2* heterozygous, lead to a classification of intermediate metabolizer in this patient (Table 2).

**Table 2:** The results of CYP2C19, ABCB1, PEAR1, and PON1 genotyping.

Locus	SNP	Allele	Results
CYP2C19^∗^2	rs4244285	G > A	GA
CYP2C19^∗^3	rs4986893	G > A	GG
ABCB1 3435	rs1045642	C > T	CC
PEAR1	rs12041331	G > A	GG
PON1 576	rs662	A > G	AA


*SNP, single nucleotide polymorphism.*

### Thromboelastogram

Thromboelastograph platelet-mapping assay was used to detect platelet reactivity of aspirin and clopidogrel by using a computerized Thromboelastograph Hemostasis Analyzer system (TEG 5000; Haemoscope Corporation, Niles, IL, United States). 1 mL of whole blood was mixed with kaolin, inverted five times, and loaded in a heparinize-coated cup containing 20 μl of CaCl_2_. TEG was started to measure AA-induced (acetylsalicylic acid-induced) and ADP-induced (adenosine diphosphate-induced) platelet aggregation. Results indicated a high drug inhibition rate of platelet (Table 3). These results lead to a higher risk of bleeding than normal.

**Table 3:** The results of thromboelastogram.

	ADP	Aspirin	Clopidogrel
DIR (aspirin: 50–90%; clopidogrel: 40–90%)	N/A	100.00 ↑	93.40 ↑
Fibrinogen level (53–72°)	74.60 ↑	63.80	69.10
Platelet function (50–70 mm)	58.50	14.90 ↓	18.90 ↓
Prediction of fibrinolysis index (0–15%)	0.00	0.00	0.00
Fibrinolysis index (0–8%)	0.00	0.00	0.00
Coagulation time (min)	22.80	8.80	7.30
Blood clot strength (4500–11000 d/sc)	7036.90	874.80 ↓	1163.30 ↓


*ADP, adenosine diphosphate; DIR, drug inhibition rate; ↓ lower than normal value; ↑ higher than normal value.*

## Discussion

Female sex is an independent factor of a higher bleeding risk than male sex during cardiac interventions. After the placement of DES, DAPT including aspirin and P2Y12 receptor antagonist is commonly used for the prevention of thrombosis. Clopidogrel, as a kind of P2Y12 receptor antagonist, remains the first-line antithrombotic drug in Asian patients post DES. The presence of inappropriate dosages of antiplatelet agents and uterine fibroids could cause AUB (Davies and Kadir, 2017). However, AUB in younger female population is often ignored or underestimated. Excessive AUB increase the risk of anemia, which could enhance ischemic heart disease. In this case, we described a 33-year-old female patient who suffered from ACS, uremia and systemic lupus erythematosus. At 6 days after PCI and DAPT with aspirin and clopidogrel, this patient complained of excessive HMB and physical discomfort for lasting 5 days. Laboratory investigations showed a reduction of RBC count, hemoglobin and hematocrit from February 26th to March 5th. A heterozygote of *CYP2C19^∗^2* lead to a intermediate metabolizer of clopidogrel. However, renal insufficiency was probably a contributing factor to an increased susceptibility to aspirin and clopidogrel. This may explain the high reactivity of aspirin and clopidogrel in TEG. Another contributing factor for excessive HMB was the use of heparin during the process of hemodialysis. This may aggravate bleeding risk in female patients when using DAPT. On the other hand, uterine myoma and cervical canal cyst may also a contributing factor for AUB.

Aspirin, in combination with clopidogrel, reduces major adverse cardiovascular events in patients with ACS managed with PCI (Yusuf et al., 2001). There are many genes influence clopidogrel response which lead to a need of pharmacogenomic testing to select antiplatelet therapy (Puymirat et al., 2012). Among those genes, *CYP2C19* genotype was well established due to the influence of clopidogrel response (Klein et al., 2018; Roden et al., 2018). The non-functional *CYP2C19^∗^2* and *^∗^3* polymorphisms impair both antiplatelet effects and bioactivation of clopidogrel (Bergmeijer et al., 2018a; Klein et al., 2018). In the acute and non-acute settings, *CYP2C19* genotyping is feasible and well used in everyday clinical practice (Bergmeijer et al., 2018b). Recently, beta-1,4-galactosyltransferase 2 c.909C>T gene variant was found which might be an independent genetic predictor of clopidogrel platelet reactivity (Pallet et al., 2018). There is still need a large sample GWAS of the function of beta-1,4-galactosyltransferase 2 in patient with clopidogrel. PEAR1, which triggers platelet aggregation, plays a very important role in platelet function. Genetic variants of *PEAR1* are associated with cardiovascular events in patients undergoing PCI and treated with aspirin in combination with clopidogrel (Nie et al., 2018; Stimpfle et al., 2018; Yao et al., 2018). *PON1* gene variations may be a risk factor of bleeding after aspirin and clopidogrel therapy (Lei et al., 2018). In this case, we tested *CYP2C19*, *ABCB1*, *PEAR1*, and *PON1* gene variations which are predictors of clopidogrel platelet reactivity. A heterozygote of *CYP2C19^∗^2* lead to a intermediate metabolizer of clopidogrel, which indicated the gene variations are not responsible for the HMB in this patient.

TEG offer a method of testing the efficiency of blood coagulation in patients with higher risks of bleeding or thrombus. The relationship between gene variation and TEG are still not clear. In Chinese patients treated with clopidogrel, polymorphisms of *CYP 2C19^∗^2* play an important role in the variability of clopidogrel’s curative effect and significantly affect the platelets inhibition ratio resulted from TEG (Sun et al., 2015). In addition, severely injured patients or patients with acute deep vein thrombosis are more hypercoagulable, which can explain the higher fibrinogen level of this patient (Spiezia et al., 2008; Differding et al., 2011). Hemodilution could also enhanced onset of coagulation (Ruttmann et al., 2006). However, the high drug inhibition rate (DIR) of aspirin and clopidogrel may resulted by using heparin during hemodialysis (Pittman et al., 2010). The high DIR of both aspirin and clopidogrel may lead to a high risk of bleeding in this patient. However, this is a patient with ACS and PCI. It is obligatory to receive aspirin and clopidogrel to avoid thrombus formation after PCI. Guideline from both Chinese experts and other countries suggested a DAPT with 100 mg aspirin q.d. po and 75 mg q.d. po clopidogrel (Levine et al., 2016; Speciality Committee on Prevention and Treatment of Thrombosis of Chinese College of Cardiovascular Physicians et al., 2018; Mehta et al., 2018).

Oxytocin is often used as a medication to facilitate childbirth. There is still lack of evidence of oxytocin being used as a hemostatic in patients with HMB. On the contrary, some evidences indicated additional of an oxytocin infusion after cesarean delivery dose not affect the overall occurrence of major obstetric hemorrhage (Sheehan et al., 2011). Furthermore, use of oxytocin drip during hysteroscopic endometrial resection did not significantly reduce operative blood loss (Shokeir et al., 2011). For the prevention of uterine bleeding after surgical evacuation of first trimester abortion, oxytocin was still not the best choice (Aramide et al., 2014).

There were several data available on how to treat HMB associated with antithrombotic agents (Maas et al., 2015; Boonyawat et al., 2017; Godin et al., 2017; Bonnington and Autry, 2018). Boonyawat et al. (2017) suggested hormonal therapy, tranexamic acid, anticoagulant management, and surgical interventions for those patients suffering from HMB. The use of oral contraceptives could significantly decreases menstrual blood loss in women with HMB (Boonyawat et al., 2017). In addition, high-dose progestin, such as norethisterone 5 mg given three times daily, was effective in reducing menstrual blood loss in women with AUB (Irvine et al., 1998). In order to reduce menstrual bleeding, oxytocin treatment was suggested by gynecologist on the fifth day of menstruation. However, 2 days of oxytocin treatment did not significantly improve HMB. Testosterone propionate and norethindrone therapy were successfully reduce HMB. Patients with AUB undergo hormone therapy have a high risk of recurrent venous thromboembolism (Martinelli et al., 2016). In this case, the patient undergoes three times of hemodialysis, which contained heparin in it. There is no need a further prevention of venous thromboembolism by heparin.

To the best of our knowledge, there was a rare report of HMB of women on reproduction age who were under antiplatelet therapy. Furthermore, there was no such case of whom has HMB with ACS, uremia and systemic lupus erythematosus. More clinical safety data on HMB of reproductive age women who are under antithrombotic therapy are required.

## Concluding Remarks

**FIGURE 2:**
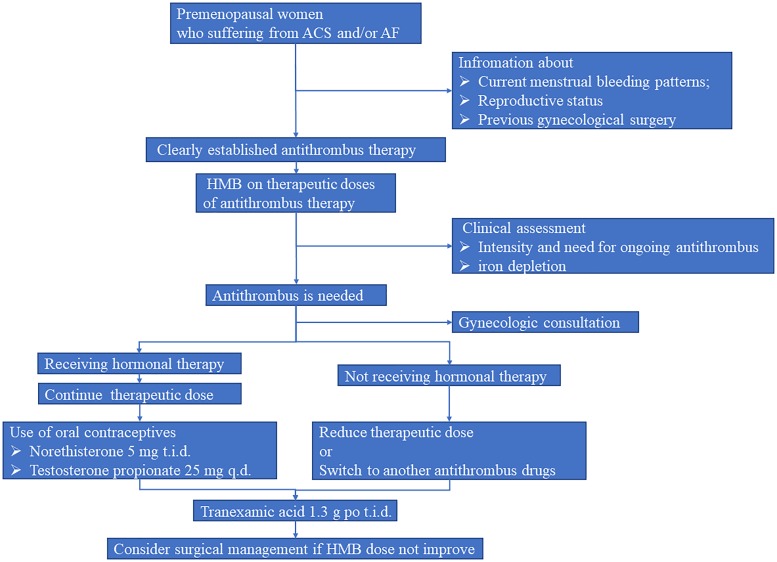
Suggested strategy for premenopausal women who suffering from ACS and/or AF. AF, atrial fibrillation; ACS, acute coronary syndromes; HMB, heavy menstrual bleeding.

As shown in Figure 2, we suggest the standard evaluation process should be clearly established in such premenopausal women. In brief, information about current menstrual bleeding patterns, reproductive status, and previous history of gynecological surgery should be asked by cardiologists or clinical pharmacists before cardiac operation. Meanwhile, gynecological examination should be performed to evaluate the presence or absence of uterine diseases. Afterward, questions concerning AUB should be carried out during the initiation of antithrombotic treatment. When patients experience HMB, hormonal therapy as well as optimal antithrombotic strategy should be conducted on the basis of menstrual blood loss and corresponding hematology testing results. Collectively, intensive management should be considered in these fragile population.

## Ethics Statement

This case report involved a human subject. Written informed consent was obtained before the preparation of this manuscript.

## Author Contributions

JZ, FW, and LS was in charge of the treatment of patient. Z-CG was responsible for collecting the patient’s information and performed the phenotyping test of *CYP2C19*, *ABCB1*, *PEAR1*, and *PON1* genotyping. F-HS was involved in the care of the patient and interpreted the results, and wrote the manuscript. HL supervised the investigations, interpreted the results, and wrote the manuscript. All authors read and approved the manuscript.

## Conflict of Interest Statement

The authors declare that the research was conducted in the absence of any commercial or financial relationships that could be construed as a potential conflict of interest.

## Footnotes

1 http://www.sino-era.com/

## Abbreviations

ABCB1: ATP-binding cassette sub-family B member 1
ACS: acute coronary syndrome
AUB: abnormal uterine bleeding
CYP 2C19: cytochrome P450 2C19
DAPT: dual antiplatelet therapy
DES: drug eluting stent
DFMH: digital fluorescence molecular hybridization
GWAS: genome-wide association studies
HMB: heavy menstrual bleeding
PCI: percutaneous coronary intervention
PEAR1: platelet endothelial aggregation receptor 1
PON1: paraoxonase 1
RBC: red blood cell
TEG: thromboelastograph
